# A Narrative Review on Advancing Pediatric Oral Health: Comprehensive Strategies for the Prevention and Management of Dental Challenges in Children

**DOI:** 10.3390/children12030286

**Published:** 2025-02-26

**Authors:** Sung-Ying Han, Chia-Lin Chang, Yung-Li Wang, Ching-Shuen Wang, Wei-Ju Lee, Thi Thuy Tien Vo, Yuh-Lien Chen, Chueh-Yi Cheng, I-Ta Lee

**Affiliations:** 1 Dental Department, Shuang-Ho Hospital, Taipei Medical University, New Taipei 23561, Taiwan; 15403@s.tmu.edu.tw; 2 Division of Pediatric Surgery, Department of Surgery, Taoyuan General Hospital, Ministry of Health and Welfare, Taoyuan 33004, Taiwan; pediatricsurgeon@mail.tygh.gov.tw; 3 School of Dentistry, College of Oral Medicine, Taipei Medical University, Taipei 11031, Taiwan; cetuswang@tmu.edu.tw (Y.-L.W.); chingshuenwang@tmu.edu.tw (C.-S.W.); 4 School of Food Safety, College of Nutrition, Taipei Medical University, Taipei 11031, Taiwan; weijulee@tmu.edu.tw; 5 Faculty of Dentistry, Nguyen Tat Thanh University, Ho Chi Minh City 700000, Vietnam; vtttien@ntt.edu.vn; 6 Department of Anatomy and Cell Biology, College of Medicine, National Taiwan University, Taipei 10051, Taiwan; ylchenv@ntu.edu.tw; 7 Department of Otolaryngology, Taoyuan General Hospital, Ministry of Health and Welfare, Taoyuan 33004, Taiwan; chuehyi@mail.tygh.gov.tw

**Keywords:** pediatric dentistry, dental development, caries prevention, oral hygiene, preventive strategies

## Abstract

Oral health is fundamental to overall well-being, especially in childhood, when dental structures and lifelong habits are established. This review provides a comprehensive analysis of children’s dental development, common oral health challenges, and evidence-based preventive strategies. Key topics include the mechanisms of tooth development, the eruption processes of primary and permanent dentition, and the management of developmental abnormalities. The prevalence, risk factors, and health impacts of dental caries in children are examined, underscoring the need for early intervention and targeted prevention. This review evaluates the effectiveness of preventive measures such as dental sealants, fluoride varnishes, and fluoride mouth rinses while highlighting the influence of dietary habits, toothbrush selection, and parental involvement on oral health outcomes. Additionally, it explores the synergistic benefits of combining preventive approaches, such as the concurrent use of dental sealants and fluoride applications, which have demonstrated superior caries prevention compared to either method alone. The effectiveness of these strategies is analyzed across different age groups, from early childhood to adolescence, with tailored recommendations for each developmental stage. Furthermore, the role of education, policy interventions, and community-based programs in addressing oral health disparities is discussed. By integrating developmental insights with epidemiological data and clinical evidence, this review provides a comprehensive framework for advancing pediatric dentistry, informing best practices, and enhancing preventive strategies to reduce the burden of oral diseases in diverse pediatric populations.

## 1. Introduction

Oral health is a critical component of a child’s overall well-being, serving as the foundation for lifelong dental health and influencing both physical and psychological development. Poor oral health in childhood can lead to pain, difficulty eating, speech impairments, and lowered self-esteem, significantly impacting a child’s quality of life and overall growth [1,2]. Despite advancements in pediatric dentistry, significant gaps remain in addressing prevalent issues such as dental caries, particularly among children aged 0–18. These disparities are even more pronounced in low-income communities, where financial constraints, limited access to specialized care, and low parental awareness create substantial barriers to effective prevention. Recent epidemiological studies have shown a significant increase in the incidence of dental caries among school-aged children. According to Huang et al., the incidence of caries in permanent teeth among children aged 5 to 14 years increased by 15.25% from 1990 to 2019, highlighting the persistent burden of this chronic condition globally [3]. High decayed, missing, and filled teeth (DMFT) scores remain persistent, with early childhood caries (ECC) and untreated cavities in primary teeth being major contributors to oral health disparities [4]. Notably, studies have shown that untreated ECC is associated with systemic complications, including growth delays and reduced weight gain, due to chronic inflammation and nutritional deficiencies [5,6].

Moreover, children with special healthcare needs, including those with developmental disabilities or chronic illnesses, encounter significant barriers in accessing preventive and restorative dental care. According to D’Addazio et al., both caregivers and dental professionals report challenges in providing adequate dental services for individuals with disabilities, citing limited specialized training, inadequate accessibility, and socioeconomic constraints as key obstacles. These disparities highlight the urgent need for targeted interventions to improve oral health outcomes in this vulnerable population [7,8]. This underscores the necessity for innovative, accessible interventions tailored to the unique needs of these vulnerable populations.

Prevention has long been recognized as the foundation of pediatric oral healthcare, with increasing emphasis on developing and implementing evidence-based strategies. Preventive measures such as fluoride varnishes, dietary modifications, and proper toothbrushing have demonstrated significant efficacy in reducing dental caries by enhancing enamel remineralization, disrupting cariogenic biofilms, and mitigating risk factors associated with poor oral hygiene [9,10]. However, the effectiveness and suitability of these methods vary by age due to differences in oral development, dietary habits, and behavioral adherence throughout childhood and adolescence. To optimize pediatric dental care, it is essential to conduct a comparative analysis of these strategies tailored to different age groups. However, many previous studies have examined these methods in isolation, without evaluating their relative effectiveness or adaptability to specific developmental stages. Furthermore, existing interventions often fail to consider the complex interplay of genetic, behavioral, and environmental factors that influence oral health outcomes. This fragmented approach limits the broader application of evidence-based strategies, particularly in addressing the diverse needs of children across various age groups and sociocultural contexts [11,12].

This review aims to address these gaps by offering a comprehensive analysis of children’s dental development stages, common oral health issues, and evidence-based preventive strategies. By examining key developmental phases, including the embryonic formation of teeth and the eruption of primary and permanent dentition, the study provides context for oral health challenges specific to different age groups. It also emphasizes the importance of implementing targeted interventions during critical stages of development [13]. Additionally, it evaluates the strengths and limitations of preventive measures, including fluoride varnishes, dental sealants, and dietary interventions, offering tailored approaches to promote optimal oral health outcomes. For example, while fluoride varnishes are highly effective in reducing caries incidence in high-risk populations, their cost and accessibility remain barriers in resource-limited settings [14,15].

Parental and caregiver involvement is also emphasized as a pivotal factor in shaping children’s oral hygiene behaviors. From fostering daily brushing habits to reducing sugar consumption, parents play a critical role in creating and sustaining healthy oral environments for children. Parental education on the importance of preventive care, such as regular dental visits and the use of fluoride products, has been shown to significantly reduce caries prevalence in early childhood [16,17]. Furthermore, policy-level interventions, such as community-based fluoride programs, school-based sealant initiatives, and public education campaigns, are highlighted as essential components of a comprehensive strategy to combat dental caries and improve pediatric oral health at a population level [18].

By integrating developmental insights with epidemiological data and preventive approaches, this review aims to enhance best practices in pediatric dentistry. It seeks to not only optimize current interventions but also guide future research and policy development. This includes advocating for longitudinal studies to better understand the long-term effectiveness of preventive strategies, as well as designing innovative technologies, such as digital oral health tools, to increase engagement and accessibility for diverse populations. Ultimately, ensuring children achieve and sustain optimal oral health throughout their developmental stages is a critical step toward reducing the global burden of oral diseases [19,20].

## 2. Key Stages of Children’s Dental Development

The development of teeth is a complex and crucial process that significantly impacts a child’s overall health and development. This begins during the embryonic stage, around the sixth week of gestation, with the formation of the dental lamina-a critical structure that serves as the foundation for future teeth. This intricate process involves dynamic interactions between ectodermal epithelium and mesenchyme, leading to the differentiation of the dental organ, papilla, and follicle. These components ultimately give rise to enamel, dentin, cementum, and periodontal ligament through a sequence of morphogenetic stages, including the bud, cap, and bell stages [21,22]. Disruptions during these stages, whether due to genetic mutations or environmental factors, can result in developmental abnormalities such as hypodontia or enamel hypoplasia, which compromise oral function and aesthetics [23].

The eruption of primary teeth, or deciduous teeth, typically begins at around six months of age and is usually completed in three years, resulting in a full set of 20 teeth. The eruption sequence follows a predictable pattern, beginning with the central incisors and progressing to lateral incisors, first molars, canines, and second molars. This process is facilitated by osteoclastic resorption of the alveolar bone and gingival remodeling, driven by local cytokines and signaling molecules such as RANKL and OPG [24,25]. Teething symptoms, including irritability, mild fever, and gingival swelling, are common during this stage and may require parental support and the use of teething aids for symptom relief. Importantly, deviations from normal eruption patterns, such as delayed eruption or premature loss of primary teeth, can signal underlying systemic or localized conditions requiring clinical intervention [26,27].

The transition to permanent dentition begins at approximately six years of age and continues into early adulthood, involving the exfoliation of primary teeth and their replacement by 32 permanent teeth. This process is driven by the pressure exerted by erupting permanent teeth, leading to the resorption of primary tooth roots and the subsequent eruption of their successors [28,29]. The sequence of permanent tooth eruption is critical for achieving proper occlusion and alignment, beginning with the first molars and central incisors, followed by lateral incisors, canines, premolars, and second molars. Delays or disruptions in this sequence, such as the impact of third molars on crowding, can lead to complications requiring orthodontic or surgical management [30,31].

Tooth eruption is accompanied by significant physiological changes, including remodeling of the alveolar bone, vascularization of gingival tissues, and adaptation of the periodontal ligament. These changes are orchestrated by molecular signals originating from the dental follicle and surrounding tissues [32,33]. Inflammatory responses during these stages, while physiological, can cause discomfort in children, underscoring the importance of supportive care and regular dental monitoring to ensure optimal development and timely intervention for eruption-related complications [34,35].

## 3. Prevention in Pediatric Dentistry

Effective prevention in pediatric dentistry is fundamental to mitigating the high prevalence of dental caries among children. This section explores key preventive strategies, providing a detailed analysis of their mechanisms, advantages, limitations, and comparative effectiveness. A summary of these strategies is presented in Table 1 to facilitate a comprehensive understanding of their clinical applications and relative merits.

### 3.1. Fluoride Applications

Fluoride-based interventions, including varnishes, gels, and mouth rinses, are cornerstone measures in pediatric caries prevention. Among these, fluoride varnish is particularly effective for younger children (ages 3–6) due to its ease of application and long-lasting protective effect, whereas fluoride mouth rinses are more suitable for school-aged children (ages 6 and above) who can rinse without swallowing. Fluoride enhances enamel remineralization and inhibits demineralization by forming fluorapatite, which is more resistant to acid erosion than hydroxyapatite [9,36,52]. Among these, fluoride varnish is highly recommended for its long-lasting effect and ease of application, typically administered every six months in dental clinics. A systematic review underscored its effectiveness in reducing caries incidence, particularly in high-risk populations [9,37]. However, professional fluoride applications can be costly, limiting accessibility in resource-constrained settings [38]. In contrast, fluoride mouth rinses offer a more cost-effective alternative suitable for home or school use. Studies demonstrate that daily or weekly use of fluoride rinses significantly reduces caries incidence in school-aged children [39,40]. Nevertheless, adherence to routine use poses challenges, and improper use may increase the risk of dental fluorosis, emphasizing the need for appropriate supervision and guidelines.

### 3.2. Dental Sealants

Dental sealants, applied to the occlusal surfaces of molars, effectively prevent decay in pits and fissures—areas most susceptible to caries in children. Systematic reviews and randomized controlled trials indicate that combining sealants with fluoride applications provides superior caries prevention compared to either method alone. A Cochrane review reported that this combination reduced caries incidence by up to 70% compared to fluoride varnish alone [43]. Similarly, a randomized controlled trial found that applying sealants followed by fluoride varnish every six months offered greater long-term protection than either treatment alone [44]. Sealants are particularly beneficial when applied to newly erupted permanent molars, making them ideal for children aged 6–14 years. Acting as physical barriers, they prevent the accumulation of food particles and bacteria. Comparative studies suggest that while both sealants and fluoride varnish are effective, sealants provide superior protection for molars with deep grooves [41,42]. However, their retention depends on material quality and application technique, requiring professional expertise. Studies indicate that resin-based sealants have an 80% retention rate after two years, while glass ionomer sealants exhibit approximately 44% retention over the same period. Higher retention rates correlate with improved caries prevention, as sealants serve as long-term barriers against bacterial colonization [53]. The Centers for Disease Control and Prevention (CDC) reports that sealants can prevent up to 80% of cavities over two years in back teeth, where most cavities occur [54]. Since sealant effectiveness is closely linked to retention, regular dental check-ups are essential to monitor their condition and ensure reapplication if necessary. Longitudinal studies indicate that sealants with high retention rates provide sustained protection against occlusal caries, reinforcing the importance of proper application techniques and follow-up care [53,54]. Despite their proven efficacy, the higher initial cost may limit widespread adoption in low-resource settings [41].

### 3.3. Dietary Modifications

Diet plays a pivotal role in caries prevention, with excessive sugar consumption being the primary risk factor. While dietary modifications are essential at all ages, early childhood (0–3 years) is a critical period for establishing healthy eating habits, and interventions during this stage have long-term benefits in caries prevention. Encouraging a diet rich in cariostatic foods, such as those containing calcium, phosphorus, and fluoride, helps in enamel remineralization and reduces caries risk [45,46]. Evidence suggests that breastfeeding, compared to bottle-feeding, is associated with a lower incidence of early childhood caries, further highlighting the importance of dietary habits during infancy [47,48]. However, promoting these changes requires sustained educational efforts targeting both parents and children, as long-term adherence remains a challenge.

### 3.4. Oral Hygiene Practices

Daily toothbrushing with fluoride toothpaste is fundamental for plaque control and caries prevention. Research indicates that powered toothbrushes outperform manual ones in plaque removal, although their higher cost can limit accessibility [49,50]. Effective brushing techniques, such as the Bass method, are crucial for maximizing benefits and avoiding damage to enamel or gingival tissues. Parent-assisted brushing for young children is strongly recommended to ensure thorough cleaning and establish lifelong oral hygiene habits [51].

### 3.5. Community-Based Interventions

Community-level programs, such as school-based fluoride mouth rinse initiatives or mobile dental clinics, address disparities in dental care access and provide preventive services to underserved populations. These cost-effective and scalable programs are particularly impactful in reducing caries prevalence among high-risk groups. For example, a school-based fluoride rinse program in Japan demonstrated significant reductions in DMFT (Decayed, Missing, and Filled Teeth) scores [18,55]. Expanding such initiatives to include dental sealants or comprehensive oral health education further enhances their public health value.

## 4. Common Dental Issues for Children

Oral health is a vital component of a child’s overall health and development, with dental caries being one of the most common oral diseases affecting children worldwide. The WHO Global Oral Health Program highlights that disparities in preventive care and education significantly contribute to the high prevalence of dental caries. This burden is particularly severe in low- and middle-income countries [56]. ECC, defined as caries in children under six years old, is a significant global concern. A systematic review and meta-analysis highlighted substantial regional variations in the prevalence of caries in primary teeth, with higher rates observed in low- and middle-income countries, reflecting disparities in oral health outcomes and access to care [57]. Dietary habits, such as frequent consumption of sugary snacks, are closely linked to the development of caries. Studies have shown that children consuming sugary snacks more than three times daily have a twofold higher risk of developing caries compared to those with lower sugar intake [46,58]. Additionally, inadequate oral hygiene practices, such as infrequent toothbrushing or the absence of fluoride toothpaste, significantly exacerbate this risk [59]. Socioeconomic disparities further compound the problem, as children from lower socioeconomic backgrounds often have limited access to dental care and education, leading to higher caries rates [60].

Another significant concern in pediatric dentistry is malocclusion, or misalignment of teeth and jaws, which varies widely in prevalence across populations and can significantly impact both aesthetics and oral function. Malocclusion affects both aesthetics and oral functions, causing difficulties in chewing, speech, and increasing trauma risk to protruding teeth. Early habits like thumb-sucking or pacifier use beyond age three contribute to anterior open bites or posterior crossbites in preschool-aged children. Mixed dentition phases in school-aged children often reveal crowding or spacing issues, requiring orthodontic intervention [61]. Gingivitis, a reversible inflammation of the gums, is a common condition among children aged 6–12. If left untreated, gingivitis can progress to periodontitis, a more severe form of gum disease characterized by the loss of attachment and supporting structures of the teeth [62]. Adolescents with systemic conditions, such as diabetes, are especially prone to early-onset periodontitis due to their compromised immune response, highlighting the need for targeted oral health interventions [63]. Addressing poor oral hygiene practices and providing education to children and their caregivers on the importance of preventive measures are critical steps in reducing the risk of these conditions and promoting long-term oral health. Dental trauma, often resulting from accidents or physical activities, is a significant issue among children worldwide. Anterior teeth are the most commonly injured, with boys being more susceptible than girls due to their higher engagement in physical activities during school-age years [64]. The absence of protective measures, such as mouthguards during sports, substantially increases the risk of traumatic dental injuries (TDIs). Proper management and timely intervention are essential to reduce complications and preserve dental function. Eruption disorders, such as delayed eruption, premature exfoliation, and ectopic eruption, often signal underlying systemic health issues, requiring careful monitoring and intervention. These disorders are often linked to systemic health issues, such as nutritional deficiencies or endocrine abnormalities. For instance, delayed eruption is frequently observed in children with hypothyroidism or Down syndrome, while premature exfoliation may indicate localized infections or conditions like hypophosphatasia [27,65]. Such disorders require careful monitoring and early diagnosis to prevent complications and support healthy dental development.

Children’s oral health challenges vary across different age groups, emphasizing the need for age-specific interventions (see Table 2 for details). During infancy (0–3 years), early childhood caries and teething pain are the most common concerns, often exacerbated by bottle-feeding habits and frequent exposure to sugary drinks [66]. In preschool years (4–6 years), malocclusion becomes more apparent as prolonged non-nutritive sucking habits, such as pacifier use, exert pressure on the primary dentition, potentially leading to anterior open bites and posterior crossbites. Additionally, inadequate brushing habits during this period may contribute to plaque accumulation and an increased risk of gingival inflammation [67]. For school-aged children (7–12 years), dental trauma becomes a significant concern as increased participation in physical activities often leads to injuries, particularly to anterior teeth, which are the most commonly affected. This stage is also marked by the transition from primary to permanent dentition, making these teeth more vulnerable to trauma. Common challenges during this phase include mixed dentition issues, such as crowding and delayed eruption, which require regular dental monitoring to ensure proper alignment and development [68]. Finally, adolescents (13–18 years) face unique challenges such as periodontal disease and orthodontic concerns, driven by hormonal changes and poor adherence to oral hygiene [69]. Understanding the prevalence and underlying factors of these common dental issues is essential for effective prevention and early intervention. Tailored approaches, such as age-specific oral hygiene programs and community-based initiatives, are critical to improving pediatric oral health outcomes. These strategies not only alleviate immediate concerns but also lay the foundation for lifelong oral health, ensuring that children reach adulthood with minimal dental complications.

## 5. Strategies to Promote Good Oral Hygiene Habits in Children

Promoting good oral hygiene habits in children is essential for the prevention of dental caries and the maintenance of long-term oral health. A combination of proper nutrition, daily hygiene practices, parental involvement, and regular professional dental care are key components in achieving optimal oral health (refer to Table 3 for a detailed summary). Recent studies have continued to highlight the critical role that diet plays in both general and dental well-being. A balanced diet rich in essential nutrients such as calcium, phosphorus, and vitamins A, C, and D supports enamel remineralization and gum health [70,71]. Emerging evidence indicates that diets with reduced sugar intake can lower the frequency of sugar spikes, thereby decreasing bacterial growth and acid production in the oral cavity, ultimately supporting better oral health outcomes [58,72]. Additionally, breastfeeding compared to bottle-feeding remains a vital protective factor in reducing the incidence of ECC, reinforcing the importance of nutritional choices during infancy [73].

Daily brushing with fluoride toothpaste is the cornerstone of effective oral hygiene practices, and recent findings emphasize its continued effectiveness. Powered toothbrushes have been shown to be more effective than manual brushes in removing plaque, especially in younger children who may lack the dexterity to properly brush with manual toothbrushes [74]. The importance of early introduction to brushing has also been supported by newer studies, which suggest that children who begin toothbrushing early are more likely to continue healthy habits into adolescence [75]. Parent-supervised toothbrushing helps ensure that young children develop good techniques, which fosters healthy oral habits that can last a lifetime. In addition to daily brushing, professional dental care, such as fluoride varnish applications and dental sealants, remains critical in preventing caries, particularly in high-risk children [9,76]. Regular dental check-ups allow for early identification of dental issues, providing opportunities for preventive treatments before significant problems arise. Recent literature also underscores the increasing importance of integrating digital tools, such as smartphone apps for oral health tracking, to further engage children and parents in routine dental care [77,78]. Several studies have identified specific digital applications, such as Brush DJ, Oral-B App, and Chomper Chums, as effective tools in improving children’s brushing habits and adherence to oral hygiene routines. These apps provide interactive features, including timers, reminders, and educational games, to encourage consistent oral care [77,78,79].

Reducing the intake of sugary snacks and drinks remains a cornerstone strategy for preventing dental caries. Frequent sugar consumption creates an acidic environment in the mouth, leading to the demineralization of enamel and promoting caries formation. A recent study highlighted the alarming rise in sugar consumption among children worldwide and its direct correlation to increased caries rates [58]. Studies show that children who consume sugary snacks more than three times a day are twice as likely to develop dental caries compared to those with lower sugar intake [46,58]. Promoting healthier alternatives, such as fruits, vegetables, and sugar-free snacks, has been identified as a viable strategy for reducing caries risk. One recent initiative has even suggested using xylitol and sorbitol-based products as substitutes for sugar, which has shown promise in reducing bacterial acid production in the oral cavity [80,81].

Fluoride continues to be a critical element in the prevention of dental caries, with its ability to strengthen enamel, making it more resistant to bacterial acids. Fluoride mouth rinses, toothpaste, and professional fluoride treatments remain effective tools in the fight against caries [9,39]. However, recent reports have raised concerns over the overexposure to fluoride, particularly in areas where water fluoridation is prevalent. It is crucial to adhere to proper guidelines to avoid the risk of dental fluorosis, a condition linked to excessive fluoride intake [82,83]. Innovations in fluoride delivery methods, such as bioactive glasses and varnishes, are emerging as effective alternatives that could provide targeted protection with reduced risks [9].

Parental involvement continues to be one of the most significant factors in establishing and maintaining good oral hygiene habits in children. Parents should model proper oral hygiene practices, including regular brushing, flossing, and healthy eating habits. Educational initiatives targeting parents, such as workshops on brushing techniques and dietary guidance, have shown to significantly improve children’s oral health outcomes by fostering a supportive home environment [84]. Moreover, recent studies emphasize the role of digital interventions for parental education, with apps providing real-time guidance and reminders to ensure that parents adhere to best oral health practices [78]. For example, the Brush Up app utilizes augmented reality (AR) to visually guide children on proper brushing techniques, while the Kolibree smart toothbrush provides real-time feedback on brushing effectiveness. These innovations have been shown to improve both parental supervision and children’s brushing performance over time [85]. Early parental support is crucial in establishing a positive attitude toward maintaining good oral health practices in children, laying the foundation for habits that last into adulthood.

In conclusion, promoting good oral hygiene habits in children involves a multifaceted approach that includes proper nutrition, effective daily hygiene practices, regular professional care, and active parental involvement. By implementing these strategies early in life, children are more likely to develop and maintain good oral health throughout their lives, reducing the prevalence of dental caries and ensuring that oral health remains a priority for years to come. These efforts are not only critical for individual children but also play an essential role in reducing the overall burden of oral diseases within communities. Continued innovation, particularly through digital tools and community-based initiatives, will further enhance these efforts and ensure sustainable improvements in pediatric oral health.

**Table 3:** Key strategies and supporting evidence for promoting good oral hygiene habits in children.

Category	Key Strategies	Supporting Evidence/Details
Nutrition	Balanced diet rich in calcium, phosphorus, vitamins A, C, D Breastfeeding over bottle-feeding	Supports enamel remineralization and gum health [70,71] Reduces incidence of ECC during infancy [73]
Daily Hygiene Practices	Brushing with fluoride toothpaste Use of powered toothbrushes Parent-supervised brushing	Fluoride toothpaste strengthens enamel [74] Powered brushes are more effective in younger children lacking dexterity [74] Early brushing fosters lifelong habits [75]
Professional Dental Care	Fluoride varnishes Dental sealants Regular check-ups	Prevents caries, especially in high-risk children [9,76] Early detection of issues allows preventive treatment
Reducing Sugar Intake	Limit sugary snacks and drinks Promote sugar-free alternatives, e.g., xylitol and sorbitol-based products	Frequent sugar consumption linked to higher caries risk [46,58] Xylitol reduces bacterial acid production [80,81]
Fluoride Application	Use of fluoride mouth rinses, toothpaste, professional treatments Innovations like bioactive glasses and varnishes	Fluoride strengthens enamel and reduces acid susceptibility [9,39] New delivery methods minimize fluorosis risk [82,83]
Parental Involvement	Modeling good hygiene practices Educational initiatives Digital tools for real-time guidance	Parents play a critical role in fostering supportive environments [84] Apps provide reminders and guidance [78]
Digital Innovations	Apps for oral health tracking and education	Engages children and parents in routine dental care [77,78]

## 6. Discussion

This review emphasizes the importance of promoting good oral hygiene habits in children as a critical factor in the prevention of dental caries and the maintenance of long-term oral health. The current findings underscore the multifaceted nature of pediatric oral healthcare, particularly in tackling prevalent issues such as ECC, malocclusion, gingivitis, and dental trauma. The complexity of these conditions requires not only individual-level interventions, but also systemic approaches, including policies and community-based programs that can influence broader health outcomes. This section discusses the clinical applications of the current preventive strategies, identifies their limitations, and suggests future directions for research, particularly the need for longitudinal studies to assess the long-term effectiveness of these interventions.

Dental caries is the most prevalent chronic disease in children worldwide, posing a significant challenge in pediatric dentistry. Beyond its clinical consequences, childhood caries place a heavy economic burden on public health systems. Global estimates indicate that oral diseases generate an economic impact exceeding $442 billion annually, including $298 billion in direct treatment costs, which account for approximately 4.6% of global healthcare expenditures [86]. Given this substantial financial impact, integrating cost-effective prevention strategies into public health programs is essential for reducing long-term expenditures on restorative and emergency dental treatments. Early intervention through fluoride applications, dental sealants, and proper oral hygiene is a well-established approach to reducing childhood caries and alleviating its clinical and economic burden on healthcare systems. Fluoride remains central to caries prevention, with fluoride varnish particularly beneficial for high-risk children. Recent studies suggest that combining fluoride with sealants provides superior protection, especially for school-aged children at elevated risk of occlusal decay. Sealants serve as mechanical barriers against bacterial colonization, while fluoride enhances enamel remineralization, creating a synergistic protective effect. A Cochrane systematic review reported that this combination reduces caries by approximately 70% compared to fluoride alone [43]. A randomized controlled trial further confirmed that children receiving both interventions had significantly lower caries incidence over three years [44]. Fluoride varnish is most effective for preschool and early school-aged children (ages 3–6), while fluoride mouth rinses are recommended for children aged 6 and older who can rinse without swallowing. However, the professional application of fluoride treatments can be costly, limiting accessibility in low-resource settings [87,88]. In many low-income communities, these challenges are further exacerbated by inadequate dental insurance coverage, workforce shortages, and geographic barriers that prevent families from accessing preventive care. Public health initiatives, such as subsidized fluoride programs or mobile dental clinics, have shown promise in addressing these disparities but remain underfunded and inconsistently implemented [89,90]. Economic evaluations indicate that school-based dental sealant programs could save up to $300 million annually by reducing the need for expensive restorative treatments [91]. Moreover, research shows that every $1 invested in preventive oral health programs, such as community fluoride interventions, yields an estimated $2 to $3 in savings by reducing future treatment costs [92]. Given these cost-saving benefits, preventive measures should be prioritized in public health policies to minimize long-term treatment expenses associated with untreated dental caries. While fluoride mouth rinses provide a more affordable alternative, adherence challenges reduce their effectiveness if not used consistently [39,93]. In low-income populations, lack of awareness and limited parental education on the importance of fluoride use further contribute to low adherence rates. Additionally, the availability of fluoride products in underserved areas is often limited due to logistical and supply chain constraints, making it difficult for families to maintain consistent preventive care at home [76]. Additionally, overexposure to fluoride, particularly in fluoridated areas, can lead to dental fluorosis, highlighting the need for proper monitoring and education on fluoride use [94,95]. Ensuring widespread access to cost-effective preventive interventions, alongside education on proper fluoride usage, is crucial for optimizing oral health outcomes and reducing the economic burden of childhood caries. In addition, the integration of emerging technologies into community oral health programs presents new opportunities to improve accessibility, engagement, and effectiveness. Digital health applications, such as mobile apps that gamify oral hygiene routines, have demonstrated success in improving children’s brushing habits and adherence to preventive care [50,77]. Teledentistry offers remote access to dental consultations, minimizing geographical and financial barriers to care. Artificial intelligence (AI)-driven diagnostic tools enhance early detection and personalized prevention strategies [96], while 3D printing facilitates the cost-effective production of oral health educational models and protective mouthguards for children [97,98]. Moreover, social media and big data analytics contribute to public awareness and data-driven policy decisions [79,94]. By leveraging these innovative technologies, community oral health programs can more effectively address disparities and promote long-term oral health benefits for children.

Dietary modifications are another key component in the prevention of dental caries. Age-appropriate dietary guidance is essential, with early childhood (0–3 years) being a crucial period for establishing sugar-reduction habits, while school-aged children benefit from structured school meal programs promoting healthy choices. High sugar consumption is strongly associated with increased caries rates, and reducing sugar intake is one of the most widely recommended strategies. However, as our findings suggest, dietary changes alone may not suffice in the long term without comprehensive parental education and community-level initiatives. Recent studies have shown that children who consume sugary snacks more than three times a day are significantly more likely to develop dental caries compared to those with lower sugar intake [58]. While promoting the consumption of cariostatic foods—such as those rich in calcium, phosphorus, and vitamin D—has proven beneficial, the challenge lies in making these dietary changes sustainable. Public health initiatives must integrate behavioral change models and use digital tools, such as mobile apps that provide real-time dietary advice and reminders for parents, to enhance the effectiveness of these strategies [99].

Parental involvement continues to be one of the most critical factors in promoting good oral hygiene habits in children. Studies consistently show that when parents are educated about the importance of oral hygiene and actively participate in their children’s oral care routines, caries rates decrease significantly [100]. The early introduction of toothbrushing, particularly with powered toothbrushes for children who may lack the dexterity for manual brushing, has been shown to foster long-term oral health habits [101,102]. However, the role of parents does not stop at education. They must also be supported by policies that provide access to dental care, particularly for children from lower socioeconomic backgrounds, who often face barriers to dental services [103]. Addressing these barriers requires coordinated efforts across healthcare systems, including the integration of dental care into general healthcare services to improve access for all families.

In terms of future research, several key areas require further exploration. One crucial avenue is the implementation of longitudinal studies to assess the long-term impact of various preventive strategies on oral health outcomes. Given the complex interplay of genetic, behavioral, and environmental factors that influence pediatric oral health, it is essential to understand how these strategies perform over time in diverse populations. Such studies would help determine the sustained effectiveness of preventive interventions such as fluoride varnish applications, dental sealants, and dietary modifications [104]. Additionally, research into innovative technologies, such as bioactive fluoride-releasing varnishes and smart toothbrushes that provide real-time feedback, could offer new avenues for more effective and personalized preventive care [105].

Another area for future research is the prevention of dental trauma, which remains a significant concern, particularly for children engaged in physical activities. Studies have shown that the use of mouthguards in contact sports significantly reduces the incidence of traumatic dental injuries [106]. The development and evaluation of preventive measures, such as mouthguards for high-risk sports, as well as further research into trauma prevention in children, is needed. This includes the incorporation of protective measures into school and community-based programs, where the risk of dental trauma is high.

Moreover, understanding the social determinants of oral health, such as the role of community-based interventions and education programs, is critical in addressing disparities in pediatric oral healthcare. Interventions that take into account socioeconomic factors and that provide access to preventive care, particularly for underserved populations, can significantly reduce the burden of dental diseases in children. Community-wide fluoride programs, school-based sealant initiatives, and parental education campaigns have shown promise in reducing the prevalence of caries in high-risk groups and should be scaled up in areas with significant oral health disparities [89,90].

## 7. Conclusions

Promoting good oral hygiene in children requires a comprehensive approach, including proper nutrition, effective daily care, regular dental visits, and active parental involvement. Given the economic burden of childhood caries, integrating cost-effective prevention strategies—such as fluoride applications, school-based sealant programs, and parental education—into national healthcare policies can reduce long-term treatment costs and establish lifelong oral health habits. However, the successful implementation of these strategies in low-income communities remains a challenge due to inadequate infrastructure, insufficient funding, and competing public health priorities. Strengthening government support, increasing financial investment, and fostering collaborations with non-governmental organizations can help bridge these gaps and enhance access to preventive dental care. Targeted preventive measures enhance effectiveness across age groups: fluoride varnish benefits young children, fluoride mouth rinses and sealants support school-aged children, and adolescents require reinforcement due to hormonal changes. Combining strategies like dental sealants and fluoride applications is particularly effective for high-risk children. Future research should focus on the long-term cost-effectiveness of these strategies, especially in resource-limited settings. Expanding school-based interventions and community fluoride programs can improve oral health outcomes while reducing healthcare costs. Additionally, advancements in preventive technologies, such as bioactive fluoride-releasing varnishes, may further optimize efficacy and accessibility, ensuring equitable oral health resources for all children.

## Figures and Tables

**Table 1:** Comparative analysis of preventive strategies in pediatric dentistry.

Measure	Mechanism	Advantages	Limitations	Applicable Age Group
Fluoride Varnish	Remineralizes enamel [9,36]	Long-lasting, effective for high-risk cases [9,37]	Requires professional application, costly [38]	3+ years
Fluoride Mouth Rinse	Continuous fluoride exposure [39,40]	Cost-effective, easy to implement [39,40]	Adherence challenges, risk of overuse [39,40]	6+ years
Dental Sealants	Physical barrier on molars [41,42]	Highly effective for deep grooves [41,42]	Professional application required [41]	6–14 years
Combination of Sealants and Fluoride	Physical barrier (sealants) + enamel remineralization (fluoride) [43,44]	Provides the greatest caries prevention, especially in high-risk children [43,44]	Higher initial cost, requires multiple visits for application and maintenance [44]	6–14 years
Dietary Modifications	Reduces sugar, promotes cariostatic foods [45,46]	Improves overall health [45,46]	Long-term adherence needed [47,48]	All age groups
Toothbrushing	Removes plaque, fluoride action [49,50]	Essential for daily hygiene [49,51]	Requires supervision for young children [51]	All age groups (parent-supervised under 6 years)

**Table 2:** Age-specific dental issues, risk factors, and prevention strategies in children.

Age Group	Common Dental Issues	Contributing Factors	Prevention and Interventions
Infancy (0–3 years)	Early childhood caries, teething pain, bottle-feeding habits, frequent exposure to sugary drinks	Bottle-feeding habits, sugary drinks [66]	Parental education on bottle-feeding and sugary drinks, early dental check-ups, introduction to fluoride toothpaste (rice grain-sized amount for children under 3 years) [66]
Preschool years (4–6 years)	Malocclusion (anterior open bites, posterior crossbites), inadequate brushing habits, plaque accumulation, gingival inflammation	Prolonged non-nutritive sucking habits (pacifier use), inadequate brushing habits [67]	Education on proper brushing habits (pea-sized fluoride toothpaste), parental supervision of brushing, monitoring non-nutritive sucking habits, early orthodontic assessments, fluoride varnish application every 3–6 months for high-risk children [67]
School-aged children (7–12 years)	Dental trauma (anterior teeth injuries), mixed dentition issues (crowding, delayed eruption), transition from primary to permanent dentition	Increased physical activities, lack of protective measures (e.g., mouthguards), mixed dentition phase [68]	Encouragement of mouthguard use during sports, regular dental check-ups, dental sealants for newly erupted permanent molars, fluoride mouth rinse (for children 6+ years), reinforcement of independent toothbrushing techniques [68]. Studies suggest that combining sealants and fluoride varnish provides superior protection against caries compared to individual treatments alone [43,44].
Adolescents (13–18 years)	Periodontal disease, orthodontic concerns, hormonal changes, poor adherence to oral hygiene	Hormonal changes, poor oral hygiene practices [69]	Tailored oral hygiene programs to address periodontal health, reinforcement of daily flossing habits, community-based initiatives for oral health education, orthodontic interventions as needed, continued fluoride application for high-risk individuals [69]
